# A Potential Application of Triangular Microwells to Entrap Single Cancer Cells: A Canine Cutaneous Mast Cell Tumor Model

**DOI:** 10.3390/mi10120841

**Published:** 2019-12-01

**Authors:** Dettachai Ketpun, Alongkorn Pimpin, Tewan Tongmanee, Sudchaya Bhanpattanakul, Prapruddee Piyaviriyakul, Weerayut Srituravanich, Witsaroot Sripumkhai, Wutthinan Jeamsaksiri, Achariya Sailasuta

**Affiliations:** 1 Biochemistry Unit, Department of Physiology, Faculty of Veterinary Science, Chulalongkorn University, Bangkok 10330, Thailand; dvmku53@hotmail.com (D.K.); Prapruddee.P@chula.ac.th (P.P.); 2 Companion Animal Cancer-Research Unit (CAC-RU), Department of Pathology, Faculty of Veterinary Science, Chulalongkorn University, Bangkok 10300, Thailand; sudchaya.bha@student.chula.ac.th; 3 Department of Mechanical Engineering, Faculty of Engineering, Chulalongkorn University, Bangkok 10330, Thailand; alongkorn.p@chula.ac.th (A.P.); t_tbenz@hotmail.com (T.T.); werayut.s@chula.ac.th (W.S.); 4 Thai Microelectronic Centre, Ministry of Science and Technology, Chachoengsao 24000, Thailand; witsaroot.sripumkhai@nectec.or.th (W.S.); wutthinan.jeamsaksiri@nectec.or.th (W.J.)

**Keywords:** canine, microfluidics, mast cell tumor, microwell, OCT4A, single cell analysis

## Abstract

Cellular heterogeneity is a major hindrance, leading to the misunderstanding of dynamic cell biology. However, single cell analysis (SCA) has been used as a practical means to overcome this drawback. Many contemporary methodologies are available for single cell analysis; among these, microfluidics is the most attractive and effective technology, due to its advantages of low-volume specimen consumption, label-free evaluation, and real-time monitoring, among others. In this paper, a conceptual application for microfluidic single cell analysis for veterinary research is presented. A microfluidic device is fabricated with an elastomer substrate, polydimethylsiloxane (PDMS), under standard soft lithography. The performance of the microdevice is high-throughput, sensitive, and user-friendly. A total of 53.1% of the triangular microwells were able to trap single canine cutaneous mast cell tumor (MCT) cells. Of these, 38.82% were single cell entrapments, while 14.34% were multiple cell entrapments. The ratio of single-to-multiple cell trapping was high, at 2.7:1. In addition, 80.5% of the trapped cells were viable, indicating that the system was non-lethal. OCT4A-immunofluorescence combined with the proposed system can assess OCT4A expression in trapped single cells more precisely than OCT4A-immunohistochemistry. Therefore, the results suggest that microfluidic single cell analysis could potentially reduce the impact of cellular heterogeneity.

## 1. Introduction

Normal and neoplastic cells are capable of varying their biology in different ways over time. This ability maintains their homeostasis, in order to cope with the dynamic changes of extracellular and intracellular microenvironments [1]. The distinguished biological setup of each cell, although they are in the same tissue, leads to the biological instability generally referred to as cellular heterogeneity. This anisotropic feature is influenced by genetic and epigenetic elements [2], microenvironments, cell-to-cell communications, and/or cell-to-acellular component interactions [3]. Such heterogeneity can confuse researchers, in terms of the real-time biology of the studied cells, and lead to unsustainable data interpretation when performing analysis using conventional methods such as immunohistochemistry, polymerase chain reactions, and microscopic morphometry, as most of these methods measure averaged biological signals from mixed cell subpopulations. Therefore, the strongest signal frequently interferes with the weaker signals produced from the rare cell groups of interest (e.g., cancer stem cells, adult stem cells, and so on). 

To resolve this impediment, single cell analysis (SCA) has been introduced throughout the world to correct for the various disadvantages of those insensitive methods [1], especially in the time-lapse monitoring of dynamic cell changes [4,5]. So far, a vast majority of biomedical engineering tools (e.g., flow cytometry, optical tweezer, laser microdissection, and microfluidics) have facilitated single cell analysis [6,7]. However, microfluidics seems to be most predominant and widely used technique. Microfluidics is an integrated science and engineering technology, which has been employed for precisely manipulating the behaviors of fluid flow in a downscale environment [8,9,10]. This method provides an attractive way to handle microparticles, as well as cells, suspended in fluid media. With typical geometric designs, biomedical and veterinary researchers can fabricate their own microfluidic devices suitable for their work. Fabrication with the transparent elastomer polydimethylsiloxane (PDMS) also supports the time-course observation of the dynamic biological alterations at optical transparencies of 240–1100 nm [11,12]. In addition, one can operate label-free cell assays, which helps to decrease the impact of cellular heterogeneity [4]. 

Large-scale immobilization is an essential step to initialize single cell analysis. Although a number of bioengineering platforms, such as optical tweezers, magnetophoresis, selective de-wetting, negative dielectrophoresis, and chemical cell patterning, are available for single cell confinement, they are low-throughput, complicated, and costly methods. The application of either external physical forces (e.g., electricity, magnets, and high-frequency optical waves) or chemical intervention may cause trapped cells to become prone to changing their biology, degenerating, or even cause necrosis. Accordingly, inertial hydrodynamic immobilization with specific microwell designs has become commonly used, instead. Its technical virtues include fabrication simplicity, high-throughput performance, low-cost fabrication, high portability, and uncomplicated system operation [13,14,15]. Numerous bioanalytical methods, such as immunocytochemistry and polymerase chain reaction (PCR), can also be combined with the microwell systems to augment their performance [13]. Furthermore, a plethora of studies have implicated the achievements of single cell entrapment with typically designed microwells so far. For example, Chen et al. showed that 70% of their 1024-microchamber microdevice was able to capture single cancer cells [16]. Swennenhuis et al. have designed and fabricated a simple array of 6400 circular microwells, with a central pore at the bottom of each, to capture single LnCAP, PC3, and SKBR-3 cell lines. The efficacy of the microdevice was approximately 67% and they could remove the targeted cells from the microdevice for further molecular biological appraisals.

Our laboratory has the aim of characterizing cancer stem cells (CSC) on the basis of self-renewal in a variety of neoplastic models, where OCT4A is the key regulator of self-renewal. Therefore, most of the researchers typically evaluate OCT4A expression. Unfortunately, our preliminary study with OCT4A-reverse transcriptase polymerase chain reaction (see Supplementary A) and OCT4A-immunohistochemistry strongly indicated expression heterogeneity. To cope with this difficulty, we fabricated a microfluidic chip containing diagonally parallel inline triangular microwells, which was able to trap single 10 μm polystyrene microbeads in our companion study. The result suggested that this microdevice was suitable for trapping single MCT cells. We also proposed that the heterogeneity of OCT4A expression would be reduced under this inertial microfluidic regime. Therefore, precise characterization of MCT cancer stem cells should be available. 

The objective of this study was to fabricate and utilize the triangular microwells to trap single primary MCT cells extracted from the real clinical specimens. The microdevice was able to capture single MCT cells without hazardous effects. OCT4A-immunofluorescence recapitulated the reduction of OCT4A expression heterogeneity, when compared to OCT4A-immunohistochemistry. Therefore, this study provides strongly evidence for the potential application of the proposed microfluidic platform for single cell analysis in veterinary research.

## 2. Materials and Methods 

### 2.1. Theoretical Background for Microchip Design

**Figure 1:**
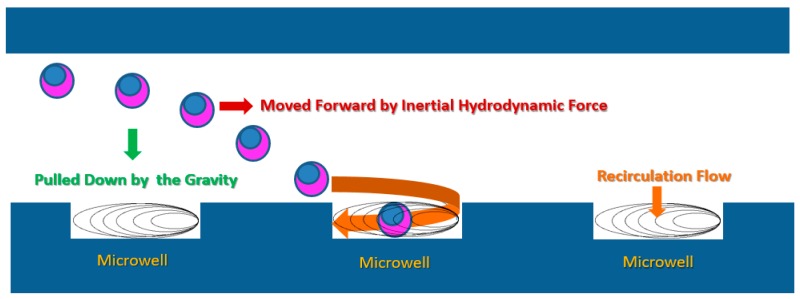
Schematic explaining the mechanism underpinning single cell entrapment by a microwell. Principally, there are three induction forces motivating the movement of an individual cell to flow into the microwell. The first is the inertial hydrodynamic force, which is responsible for moving the cell along the main flow axis. The second is the gravitational force, which attracts the cell to move across each layer of the laminar flow. Consequently, the cell is pulled down to the floor of the microdevice, where the microwell array exists. The last is recirculation, which induces the cell to flow into the microwell (modified from Park et al., 2010) [17].

The microdevice was designed based on the predilect parameters previously described by Park et al. [17]. In principle, while the cells are floating in the media, gravitational force pulls the cells across the axial streamlines and down into the base of the microdevice. Whenever an individual cell is close to a microwell, the recirculation force generated by the change of fluid momentum in the microwell will direct the nearest cell into the microwell (Figure 1).

To comprehend how the microwell geometry affects recirculation, we systematically performed computational simulations using the COMSOL Multiphysics^®^ version 5.3 software (COMSOL, Los Angelis, CA, USA). The setup parameters were similar to those formerly described in our companion study. Briefly, the simulation compartments consisted of microwells on the basal layer and a 1 mm (length) × 80 μm (width) × 160 μm (height) rectangular main flow channel. The microwells were assigned to have a 40 μm side length (equivalent triangle), 40 μm diameter (circular), or 40 μm perimeter (square microwells), with 15 μm depth in all simulated microwells. The computational domains were symmetric on both sides, with non-slip boundaries. This condition did not affect the streamline profiles of the simulated microwells located near the edges of the microdevice, according to the fully-developed boundary layer. The finite element method was utilized in the simulation. The total number of meshes for each object was 3–5 × 10^6^. The media was water, with a density of 1000 kg/m^3^ and viscosity of 0.001 N/m^2^. The flow condition was uniformly laminar and the flow module was single-phase. The velocity was confined to 0.1 mL/min [18]. 

**Figure 2:**
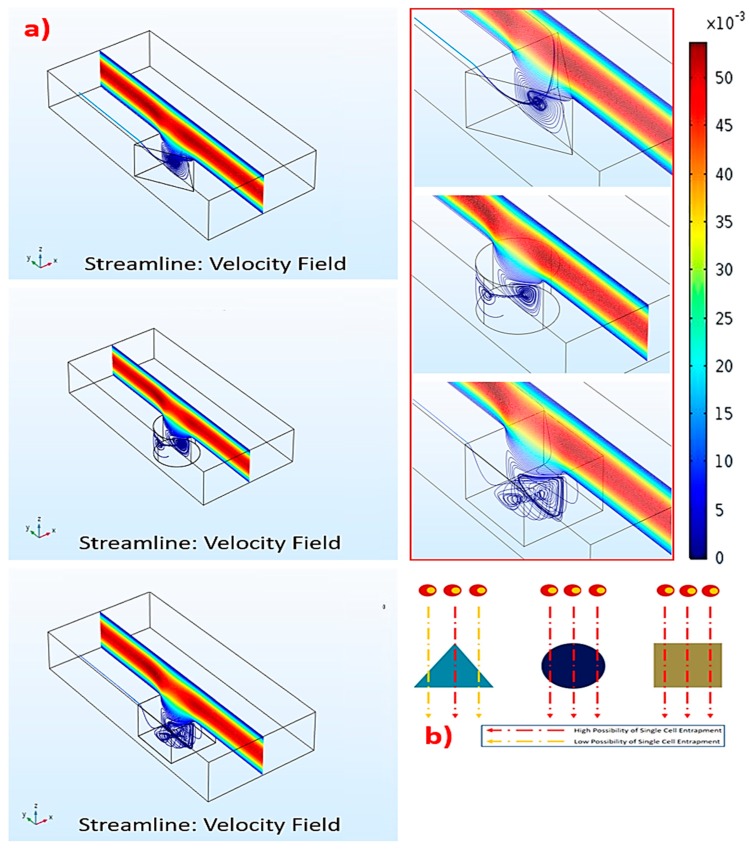
Computational fluidic dynamics (CFD) of the recirculation: (**a**) The formation and the velocity field of recirculation flow for each microwell type, and (**b**) the effect of microwell geometry on the probability of single cell entrapment.

The results uncovered that the strongest recirculation originated in the triangular microwell, while the vorticities in the circular and square microwells were weaker (Figure 2a). Thus, the triangular microwell provided the highest possibility to trap single cells (Figure 2b). As recirculation in the triangular microwell occupied a vast area, extending from the back to the front, this would accommodate the trapped cells to locating in the microwell. On the contrary, recirculation in the circular and square microwells were smaller and covered some parts of the microwells, causing the upper mainstreams to flow deeply into these microwells. Such deep flows could generate flow disturbances, leading to a random trapping manner with a smaller possibility of single cell trapping.

In fact, two vortices occurred in the triangular microwell. One was a pair of counter-rotating vortices at the leading edges of the microwell. The other one was a spanwise vortex, which covered the upper layer of the microwell. This was due to the main flow over the microwell. The vortex rolled from the back to the front after colliding with the trailing wall. Together, these two vortices created the other pair of vortices located inside the microwell. These secondary vortices could disturb the cells and reorganize the trapping manner, such as single, double, or multiple. Furthermore, the extent of these vortices depended on the size of microwells, causing the size-based selection of microwells for the suitable cells. For instance, 40, 60, and 80 μm triangular microwells would be suitable for 10, 15, and 20 μm cells, respectively. However, this unique flow characteristic was not generated in the square or circular microwells. 

**Figure 3:**
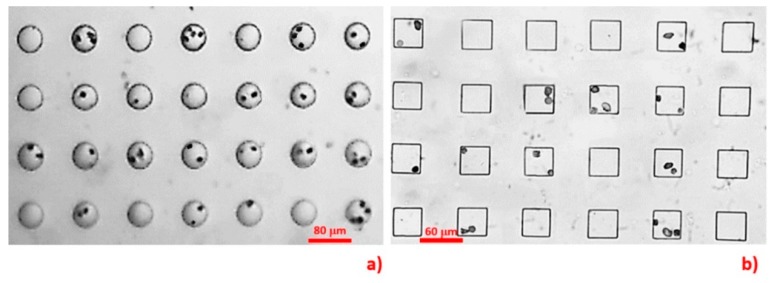
This figure recapitulates the low possibility of the circular (**a**) and the square microwells (**b**) to trap Giemsa-stained single MCT cells. Notably, the data were obtained from our unpublished preliminary study.

In our preliminary study (unpublished data), we fabricated parallel inline circular and square microwells (Figure 3) to testify the computational results. The microdevices were fabricated and instrumented with the same protocols detailed in Section 2.1 and Section 2.3, respectively. The dimensions of the microwells were equal to those defined in the simulation. The MCT cells in that study were harvested by a similar procedure as that described in Section 2.5 and stained with Giemsa dye. The results indicated that 55% of the circular and 42% of the square microwells occupied the cells. Both microwell geometries had low efficiencies in trapping single MCT cells. The single-to-multiple entrapment ratios were 0.59:1 for the circular microwells and 0.22:1 for the square microwells.

### 2.2. Microdevice Fabrication and Characterization

The configuration of the microfluidic device was composed of two functioning layers. The first layer was comprised of an array of 15 μm (depth) × 40 μm (side length) equilateral triangular microwells lined in a matrix of 63 × 143 microwells. This dimension was selected as the size of the MCT cells we used was on the scale of 10 μm. All microwells were embossed on the floor of the microdevice. Each inline microwell was diagonally parallel to the adjacent ones. This alignment was different from the original described in the study of Park et al., and the diagonally parallel orientation showed superior performance for trapping 10 μm polystyrene microbeads in our companion study [18]. It increased the possibility that cells could arrive at an individual microwell, as there was no blockage of cell movement from the upstream microwells. Therefore, the trapping efficiency could be improved. 

**Figure 4:**
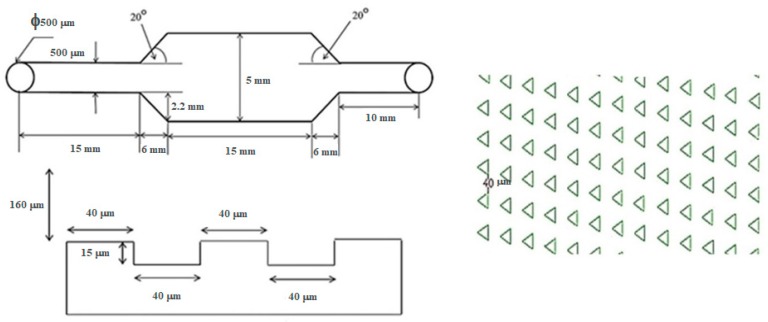
Blueprint of the designed microdevice. The first layer is the array of 40 μm high × 15 μm deep equilateral triangular microwells. The alignment is in a matrix of 63 × 143 diagonally parallel inline-triangular microwells embossed on the floor. The second layer is the 160 μm-high main flow microchannel, which covers the microwell array.

**Figure 5:**
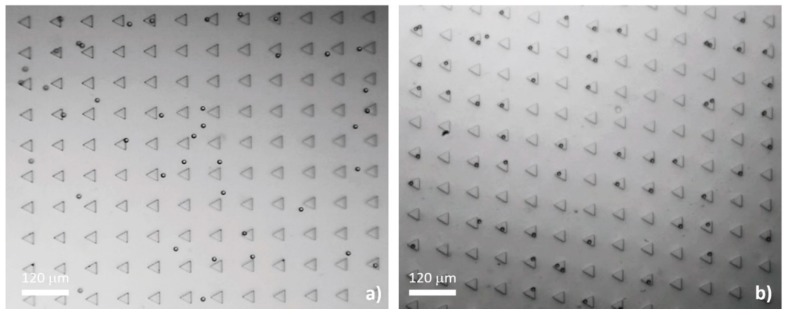
Comparison of the trapping efficacy between the straight inline-triangular microwells (**a**) and the diagonally parallel inline-triangular microwells (**b**). The trapping performance is low in the straight inline-triangular microwells, while the efficiency is increased with the diagonally parallel inline-triangular microwells.

The second layer of the microdevice was 160 μm (height) × 27 mm (length) × 5 mm (width), where the main flow microchannel covered the whole area of the microwell array. Figure 4 illustrates the blueprint of the microdevice and Figure 5 depicts the comparative performance of the parallel and the diagonally parallel inline-microwells in trapping 10 μm polystyrene microspheres, from our companion study. 

The microchip was fabricated using photoresist standard soft lithography. Namely, the blueprint of the microchip was drawn using a commercial computer-aided-design software (AutoCAD^®^ 2016, AutoDesk, San Rafael, CA, USA). A 6 inch silicon wafer was cleaned with piranha solution for organic contaminant removal. The wafer was spin-coated with hexamethyldisilazane (HMDS) at 1000 rpm and then baked at 90 °C for 90 s. The photoresist film PFI-34a (Sumitomo, Tokyo, Japan) was spin-coated onto the wafer at 1000 rpm for 20 s, with a final thickness of 2 μm. The pattern of each layer was then transferred onto the wafer with 365 nm ultraviolet exposure at an intensity of 40 mW/cm^2^ through the photoresist mask for 5 s. The patterned wafer was cured at 110 °C for 100 s to harden the surface and was then developed in SD-W (Sumitomo, Tokyo, Japan). The wafer was chemically etched using deep reactive ion etching (DRIE) with gaseous sulfur hexafluoride (SF_6_)/octafluorocyclobutane (C_4_F_8_). Replicas of both layers were fabricated with liquid elastomer polydimethylsiloxane (PDMS) (Sylgard™ 184, Dow Corning, Midland, MI, USA) and both layers were assembled together with oxygen plasma in the conditions of 30 W of 40 sccm oxygen for 90 s. 

**Figure 6:**
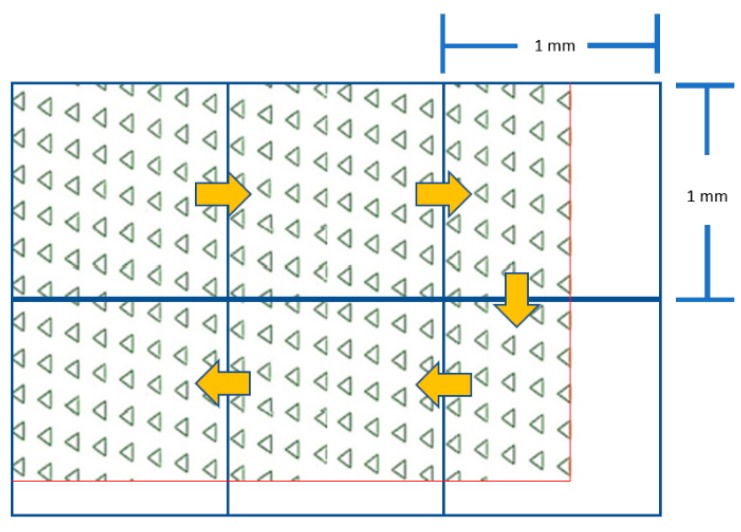
The grid-based counting method. The yellow arrows indicate the counting direction. The red lines represent the boundaries of the microwell array. Remarkably, the drawing is not to scale.

The 1 mm^2^ transparent grid sheet was underlaid under the microdevice as a reference frame. Then, the total number of microwells was counted, frame-by-frame, throughout the whole area of the microdevice (Figure 6). This technique was applied for numbering the occupancy rate, as well as the viability of the trapped MCT cells.

### 2.3. Microdevice Instrumentation and Experiment Setup

**Figure 7:**
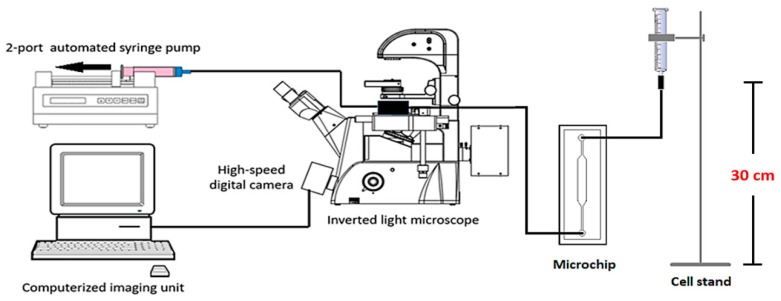
The experimental setup used in this study. The microdevice is connected with the two-port automatic syringe pump at its outlet and the cell suspension is fed through the inlet port. Single cell entrapment and OCT4A-immunofluorescence were monitored with a PE filter and a reverse light microscope, respectively.

The concentration of MCT cell suspension used for each experiment round was 1 × 10^5^ cells/mL. The microchip was connected to the cell delivery system by the inlet port, while its outlet was connected to a two-port automatic syringe pump (F100, Chymex, Stafford, TX, USA). This pump reversely empowered the microdevice to pull the cell suspension into the microchannel. The flow rate was set to 0.1 mL·min^−1^ for 30 min. Every 10 minutes, we periodically paused the flow for 3 min to allow the cells to sediment by gravitation, for increased trapping performance. The entrapment was visualized under light microscopy. The experiment was performed in triplicate, with a new microdevice used for each round. The efficacy of each microfluidic device was recorded in terms of the total numbers of microwells, cell-occupied microwells, single cell occupancy, and multiple cell occupancy. The average of each parameter, as well as its standard deviation, was calculated. Figure 7 illustrates the schematic of the microdevice and instrumentation used in this study.

### 2.4. Specimen Collection

Six MCT patients were selected from the oncology unit, Small Animal Teaching Hospital, Faculty of Veterinary Science, Chulalongkorn University (Bangkok, Thailand). Each case underwent mass excision and 30 g of its mass was manually dissected to remove adipose tissues. The samples were then halved for use in OCT4A-immunohistochemistry and microfluidic-based single cell assays, respectively. This protocol was approved by Chulalongkorn University animal ethic committee (Reference No. 1631055) and all animal clients were consented in the usage of MCT specimens.

### 2.5. Single MCT Cell Isolation

To isolate a cluster of fresh single MCT cells, the remaining half of the MCT tissue from each case was chopped into small pieces and pooled together in a 2 mL centrifuge tube (Corning, Corning, NY, USA). They were trypsinized with 1 mL of 0.025% trypsin (Sigma-Aldrich, St. Louis, MI, USA), admixed with 0.01% EDTA (Thermo Fisher Scientific, Waltham, MA, USA) in 2 mL of 1× PBS at 38 °C for 20 min, and the reaction was then terminated with 2 mL of 2% fetal bovine serum (Gibco, Life Technology, Carlsbad, CA, USA). Single MCT cells were segregated from indigested tissue remnants and auto-aggregating cell spheroids by percolating through a 40 mm cell strainer (Falcon, Corning, NY, USA). They were washed with 1× PBS and centrifuged at 3000 rpm at 4 °C for 5 min twice. The cells were cultivated with RPMI-1640 (Gibco^®^, Thermo Fisher Scientific, Waltham, MA, USA) with 10% FBS for 4 h to enhance their viability.

### 2.6. Viability Evaluation

Briefly, a mixture of 500 μL of 0.4% Trypan blue (Hyclone™, GE Healthcare Life Sciences, Marlborough, MA, USA) and 200 μL of 1× PBS was prepared as the working solution. The assessment of cell viability was carried out for the MCT cells after trypsinization from the masses and in the trapped cells. For trypsinized cells, 300 μL of the cell suspension was aliquoted into a new 2 mL collection tube and incubated with the working solution for 5 min in a dark chamber. Thereafter, the cells were loaded to a hemocytometer. The stained versus the unstained cells were counted. 

For trapped MCT cells, the media in the microchannel was drained out and its space was slowly flushed with working solution. All cells were incubated with the solution for 10 min in a dark cabinet. Data acquisition was performed with light microscopy in the whole area of the microwell array. Cell viability was reckoned as the percentage of viable cells using the following equation: *C_v_* = 100(1 − *Ci*/*N*), where *C_v_* is the percentage of viable MCT cells, *C_i_* is the number of inviable MCT cells, and *N* is the total number of either single MCT cells in the cell suspension or trapped cells. 

### 2.7. OCT 4A-Immunohistochemistry

The process was slightly modified from a standard protocol, previously described by Webster et al., 2007. Stepwise, 4 μm formalin-fixed-paraffin-embedded (FFPE) MCT sections were deparaffinized and rehydrated with xylene and graded alcohols, respectively. Afterward, the sections were incubated with citrate-buffered saline (pH = 6.4) for 5 min and microwaved for 15 min to retrieve OCT4A antigens. The endogenous peroxidase activity was terminated with hydrogen peroxide (H_2_O_2_) at room temperature for 30 mi. Then, the non-specific proteins were blocked with 1% bovine serum albumin (BSA) at 37 °C overnight. The OCT4A was then immunolabelled with mouse anti-human OCT4 monoclonal antibodies (Clone 40/Oct-3, Becton and Dickinson, Franklin, NY, USA) at a concentration of 1:100 at 37 °C in a dark humidified chamber overnight. Ultimately, the EnVision™ peroxidase system (Dako Denmark, Hovedstaden, Denmark), with 3,3′-diaminobenzidine tetrahydrochloride (DAB) as the chromogenic substrate, was utilized to colorize the labeled OCT 4A. The reaction was terminated with EnVsion™ FLEX Peroxidase-Blocking Reagent (Manufacturer, City, Country). The sections were rinsed with 1× phosphate-buffered saline (PBS) for 10 min, in order to eliminate chemical residues. Soon after, the nuclei were counterstained with Meyer’s hematoxylin for 1 min. All tissue sections were cleaned with running tap water for 5 min and then rehydrated with backward graded alcohols. The immunopositivity of OCT4A in the nuclei was visualized under light microscopy [19].

### 2.8. OCT4A-Immunofluorescence of Microfluidic-Entrapped MCT Cells

Stepwise, the fluid media in the microdevice was flushed out. Cell membranes were permeated with membrane-piercing solution (CU-Vet MPS^®^, Chulalongkorn University, Bangkok, Thailand) at the ambient temperature for 30 min. The trapped cells were rinsed twice with 1× PBS and further incubated with 2% fetal bovine serum at room temperature for 30 min. Later, they were incubated with PE-conjugated mouse monoclonal anti-human OCT4 antibodies (Clone 40/Oct-3, Becton and Dickinson, Franklin Lakes, NY, USA) at a dilution of 1:100 at 37 °C for 2 h. Their nuclei were stained with 4′,6-diamidino-2-phenylindole (DAPI) for 10 min in the dark chamber. Ultimately, red fluorescence signals in the nuclei were detected using an inverted fluorescence microscope with a phycoerythrin (PE) filter. Finally, the result was compared to OCT4A-immunohistochemistry.

## 3. Results

### 3.1. Microdevice Fabrication and Geometric Feature

**Figure 8:**
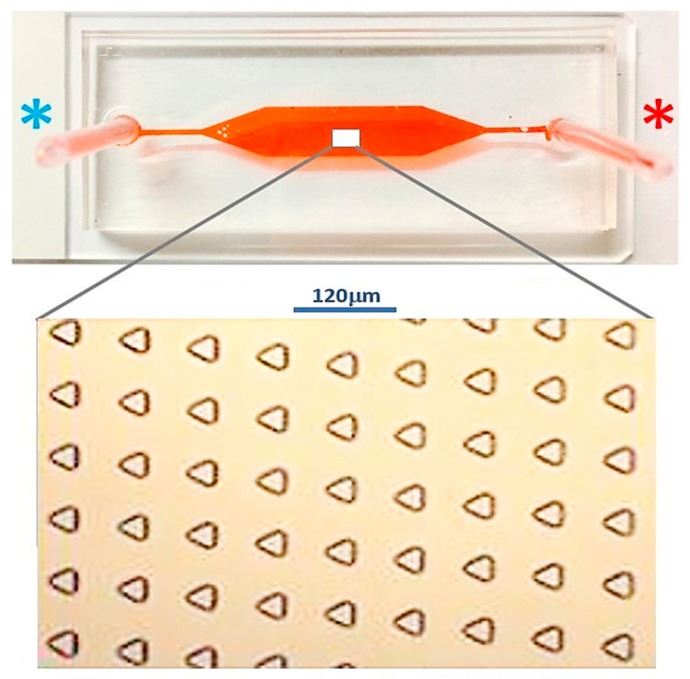
The external appearance of the fabricated microdevice consists of the inlet (**blue asterisk**) and the outlet (**orange asterisk**). The lower inset exhibits the internal configuration of the inline-microwell array.

The microdevice for single MCT cell entrapment was fabricated using PDMS. The two-layered composite consisted of the ground layer and the upper layer, which served as the main flow microchannel. The average number of the microwells was 9310 in the total lining, in an array of 63 × 143 microwells, as determined in the blueprint. The geometry of each microwell was an equilateral triangle with 40 μm side length and 15 μm depth. The microwell array was encompassed by a 27 mm long, 5 mm wide, and 160 mm high main flow microchannel. The length of the cell entry microchannel was 15 mm, which was connecting to the inlet port; meanwhile, the length of the cell exit microchannel was 10 mm, which was linked to the outlet port. The difference in the lengths of the entry and the exit microchannels did not impact the hydrodynamic profile, despite a very low inflow rate. The features of the microdevice are shown in Figure 8.

### 3.2. Microfluidic Single MCT Cell Entrapment

**Figure 9:**
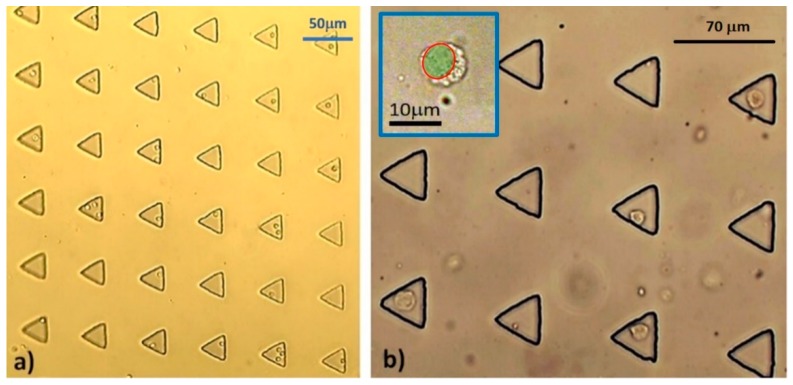
The entrapment of single MCT cells in the triangular microwells under different power fields: (**a**) At the medium-power field (1×) and (**b**) at the high-power field (40×). Although most of the microwells captured single MCT cells, multiple cell entrapment was observed in some microwells as well. Noticeably, the biological configurations of all trapped MCT cells were normal. The plain individual MCT cells were round cells with concentric nuclei and granularly clear cytoplasm. The blue inset illustrates the magnified morphology of a single MCT cell under plain light microscopy. The green shading indicates the nuclear space.

The viability (*C_v_*) of trypsin-isolated MCT cells was found to be 83%. At the designated inflow rate, the result substantially demonstrated that most of the individual triangular microwells effectively trapped single MCT cells. On average, the occupancy rate of the microwells was 53.1% (4949 out of 9310 microwells). Of these, 3614 microwells (38.82%) captured single MCT cells, while 1335 microwells (14.34%) contained multiple MCT cells. The ratio of single to multiple cell occupation was, thus, around 2.7:1. The average *C_v_* of trapped cells was 80.5%. Table 1 summarizes the propensity of MCT entrapment in the microdevice at each round. The feature of viable trapped MCT cells in the microwells is shown in Figure 9.

### 3.3. Immunohistochemistry of OCT4A Isoform

**Figure 10:**
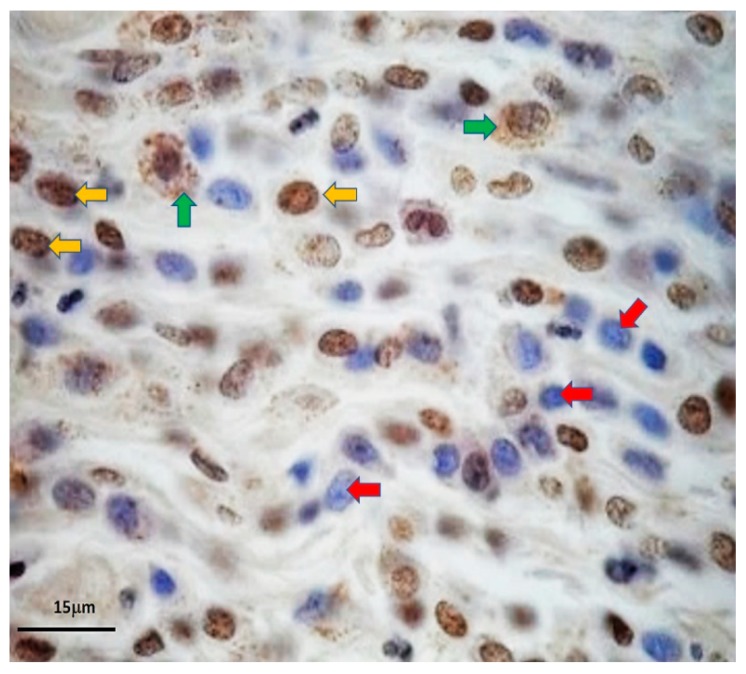
OCT4A-immunohistochemistry uncovered the activity of the key embryonic transcription factor OCT4A in self-renewing MCT cells. The positivity was in the nuclei of the putative MCT cancer stem cells (**yellow arrows**). However, immunopositivity was also observed simultaneously in the cytoplasm of such MCT cells (**green arrows**). On the contrary, the negative cells were stained with Mayer’s hematoxylin in their nuclei only (**red arrows**).

Evidently, the OCT4A-immunohistochemistry of MCT uncovered two prominent staining patterns. The first format was the intranuclear staining, in which the immunopositivity was in the nuclei of the MCT cells. This pattern reflected OCT4A isoform expression, according to the interpretative criterion. The latter scheme referred to cytoplasmic staining, which exhibited that OCT4A-immunolabeling substantially dispersed throughout the cytoplasm. Meanwhile, the negative cells were only counterstained with Meyer’s hematoxylin. Figure 10 exhibits the positive and negative OCT4A-immunohistochemistry in MCT cells.

### 3.4. OCT4A-immunofluorescence of Trapped Single MCT Cells

**Figure 11:**
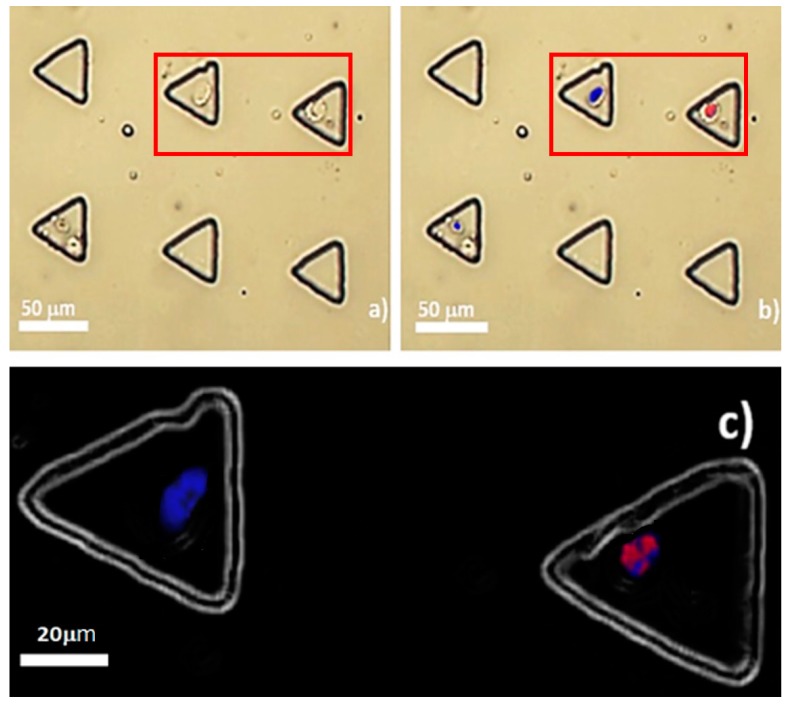
OCT4A-immunofluorescence of trapped MCT cells: (**a**) Plain background at the locations with OCT4A-immunopositivity. The cells are round-to-oval with large nuclei. The cytoplasm was scarce and seemingly clear. The membrane boundary was distinctly defined; (**b**) intranuclear immunopositivity of OCT4A in an trapped MCT cell. After background merging, the positive cell was in its corresponding microwell; and (**c**) OCT4A fluorescence signal in the nucleus of the positive cell in the red box, compared to the negative subject (stained with DAPI only). Notably, the background was converted to a dark shade for the purpose of visual clearness.

To address the reduction of the anisotropic OCT4A expression under microfluidic single cell analysis, OCT4A-immunofluorescence was combined with the microfluidic system. Positivity was presented in very few trapped MCT cells. The nuclei of those positive cells were colored red, whereas negative MCT cells were not tangible and were intranuclearly stained with DAPI only (Figure 11b,c). There was no positivity in the cytoplasm, as in case of OCT4A-immunohistochemistry. On average, 2 out of 4949 occupied microwells (0.0404%) contained single positive MCT cells. There was no OCT4A-immunopositivity observed in the multiple-occupied microwells in all rounds of the experiments. As the theoretical frequency of CSC in most types of neoplasms is in the range of 1 × 10^−6^ to 1 × 10^−2^, this result roughly suggests the rarity of cancer stem cells in MCT [3,20,21,22,23].

## 4. Discussion

This paper has reported, for the first time, the potential application of a triangular microwell array to trap single primary cells harvested from clinical specimens. The study outcome also increases our comprehension of the recirculation generated, as well as its effect on the primary cells. In accordance with the results, we have exhibited the potential application of our triangular microwells for single MCT cell entrapment. A microchip was fabricated with the elastomer PDMS under standard soft lithography. Obviously, single MCT cell trapping took place in a stochastic manner and depended on the amplitude of recirculation in the microwells and the stationary time of cells in the main flow microchannel before considering the gravity effect. The performance of the microdevice was still high-throughput when compared to the typical active methods. Within 30 min at the slow flow rate of 0.1 mL·min^−1^, the capture rate was 53% on average with low standard deviation. The ratio of single to multiple cell occupation was around 2.7:1. This microfluidic platform was uncomplicated and easily instrumented and there was no need for well-trained personnel to set up and operate the system. In spite of trypan blue assay, the results consistently showed a high level of cell viability after single cell entrapment. However, the viability of the trapped MCT cells seemed to be slightly reduced from the start, as not all MCT cells were trapped in the microwells. However, further viability assessments may indicate that our microfluidic platform is cell-friendly and that the inertial hydrodynamic factors in the system are not pernicious. Fundamentally, the deleterious effects of a microfluidic system depend on the designed geometry [7], the intrinsic hydrodynamic mechanisms used, and the cell type. For example, the inertial hydrodynamic forces have been shown to be detrimental to sorted MCT cells, but not to canine leukocytes, in the case of size-based cell separation with a spiral microchannel [24]. 

Although our microfluidic device is not all new and the performance of our microdevice was still lower than the others using the similar platforms, there were some common positive traits. The major similarity is that the triangular microwells utilize inertial hydrodynamics and gravitation to achieve single cell capture. The other similarity is the dimensions of the triangle (i.e., the side length and the depth), which determines the size-based matching between cells and microwells. In addition, all triangular microwell regimes were not lethal to the cells used in the experiment. 

On the other hand, there were also points of difference for our microdevice, when working with the primary cells obtained from fresh MCT specimens. Their dynamic biophysical properties (e.g., pleomorphic shape, anisotropic size, cell density, and so on) might differ from those in the cell lines that are rather unique. These heterogeneous properties may have governed the efficacy of our microdevice. For example, low-density MCT cells may not be as influenced by gravity and, instead, float out the microdevice. Large MCT cells, per se, may not fit the size of the microwells. The other factor is the time in which MCT cells have been left in the microchannel. Prolonged stay times in the main flow microchannel may allow gravity to completely pull down the cells into the microwells. The effect of the gravity has been distinctly demonstrated by Tu’s group study. In that study, they fabricated triangle microwells to trap single HeLa and human gall bladder carcinoma SGC-996 cells. They allowed the cells to stay in the microchannel and gradually drop down into the triangle microwells by gravity. Even though the configuration of their microdevice was similar to ours, the mechanism of single cell trapping was quite different. Their microdevice had to be flipped after cell loading to allow gravity to pull the cell down, while our microdevice utilizes both recirculation and gravity. They also enhanced single cell trapping with protein-based cell patterning, whereas our microfluidic platform is label-free. These different parameters, of course, may affect the efficacy of single trapping. 

Upon the cancer stem cell hypothesis, the taproot of oncogenesis are the cells referred to cancer stem cells. However, the typical biology of this cell species has not yet been fully determined, due to cellular heterogeneity. However, consensual biological methods have been established for CSC characterization. The first is that this cell species can replenish itself through the specific biological process called self-renewal, which is generally modulated by the embryonic transcription factor OCT4A [25,26]. The other is that the population of cancer stem cells in a given neoplasm is quite rare [27,28]. Upon OCT4A-immunofluorescence under our microfluidic regime, the result substantially reinforced that the down-scale environment of microfluidics did not interfere but, to the contrary, enhanced the performance of the method. Few MCT cells were positive for the immunoassay, these were supposed to be the putative MCT cancer stem cells. Positivity was detected only in their nuclei without the background noise in the cytoplasm. This observation was very clearly contradictory to OCT4A-immunohistochemistry, both in this study and in previous studies in which positivity was seen both in the nuclei and the cytoplasm [19]. This may indicate that many positive signals of OCT4A under tissue-based methods are artefactual, as OCT4A signal noises in the cytoplasmic compartment of MCT cells were eradicated under our microfluidic platform. This could address the controversy in the rarity of MCT cancer stem cells, as well. Despite only 0.04% of trapped MCT cells expressing OCT4A, the results might suggest the scarcity of putative MCT cancer stem cells, in contrast to the result from OCT4A-immunohistochemistry. Taken altogether, our study implies that OCT4A-immunofluorescence under our microfluidic platform is more reliable than the conventional methods including OCT4A-immunohistochemistry for the validation of the existence of putative cancer stem cells in MCT. 

However, there was still a likelihood that our triangular microwells captured multiple MCT cells; therefore, this flaw also requires correction, in order to increase the system efficacy, before this technology can serve as a major tool for single cancer cell analysis in veterinary medicine.

## 5. Conclusions

Cell heterogeneity is a serious problem, which hampers our understanding of real cell biology. To overcome this issue, single cell analysis is a conceptual solution which has been adopted worldwide. Although there are many methods and technologies which can be applied for single cell analysis, microfluidics seems to be the most outstanding. Regarding our current study, computational fluid dynamic analysis has shown that potent recirculation is generated by triangular microwells. To this end, a microdevice consisting of an array of diagonally parallel inline triangle microwells, as well as a main flow microchannel, was fabricated with the elastomer PDMS under standard soft lithography.

Our microfluidic platform has the capacity to trap single MCT cells at a high-throughput rate; furthermore, the system was not detrimental to the cells, according to trypan blue assessment. Importantly, the problem of immunostaining background noises has been resolved with our microfluidic single cell analysis. Merged OCT4A-immunofluorescence has indicated no OCT4A-immunopositivity in the cytoplasm of single MCT cells trapped in the microwells, when compared to OCT4A-immunohistochemistry. 

Accordingly, our microfluidic system has the potential to alleviate the problem of anisotropic OCT4A expression in primary MCT cells and, as such, it is highly suitable for single cell analysis in veterinary research. However, the influences of the design parameters (i.e., microwell dimension, microwell alignment, stay time of cells in the microchannel, flow rate in the main microchannel, and intrinsic hydrodynamics) require careful evaluation, both at the computational and experimental levels, for improving the efficacy of the microdevice.

## Figures and Tables

**Table 1:** The overall performance of each microdevice. On average, 4949 microwells (53.1%) trapped MCT cells. Of these, 3614 microwells (38.82%) captured single MCT cells, while 1335 microwells (14.34%) contained multiple MCT cells.

Type of Occupancy	Microdevice I	Microdevice II	Microdevice III	Average ± SD
Total fabricated microwell	9309	9313	9308	9310 ± 2.65
Occupied microwell	4951	4962	4934	4949 ± 14.11
Single cell occupancy	3605	3602	3635	3614 ± 18.25
Multiple cell occupancy	1346	1360	1299	1335 ± 31.95

## Supplementary Materials

The following are available online at https://www.mdpi.com/2072-666X/10/12/841/s1, Figure S1: The 405 bp amplicons of OCT4A.
