# Supplementary material for: A Potential Application of Triangular Microwells to Entrap Single Cancer Cells: A Canine Cutaneous Mast Cell Tumor Model
Source: Micromachines (Basel). 2019 Dec 1;10(12):841. doi: 10.3390/mi10120841 (PMC6953038; doi:10.3390/mi10120841)
Supplement: Supplementary file 1 [file micromachines-10-00841-s001.pdf]

# Supplementary Materials: A Potential Application of Triangular Microwells to Entrap Single Cancer Cells: A Canine Cutaneous Mast Cell Tumor Model

**Dettachai Ketpun, Alongkorn Pimpin, Tewan Tongmanee, Sudchaya Bhanpattanakul, Prapruddee Piyaviriyakul, Weerayut Srituravanich, Witsaroot Sripumkhai, Wutthinan Jeamsaksiri, and Achariya Sailasuta ***

**The Preliminary Result of OCT4A reverse transcription-polymerase chain reaction (RT-PCR) illustrates cellular heterogeneity in Mast Cell Tumor (MCT) cells**

Isolation of the oncogenic headwater cells in a given neoplasm is an indispensable process. Undeniably, the outright comprehension of the biology of these cells will determine the right way to eradicate the disease completely in the future. Nowadays, much more research has suggested the existence of a typical group of neoplastic cells answerable for tumorigenesis and disease propagation. Their functionality is basically stemness-dependent, and the most importance is self-renewal. This discovery has led to the establishment of a state-of-the-art oncogenic model referred to cancer stem cell hypothesis. Fundamentally, this assumption states that not all neoplastic cells in a neoplasm are equivalently capable to replenish themselves. But only a small fraction called cancer stem cells exclusively possesses this competence by which it is functioning through the asymmetrical cell division. Nevertheless, this ability can virtually be compatible to the self-renewal of embryonic stem cells of which it is majorly regulated by the key embryonic transcription factor OCT4 encoded by the gene OCT4.

Paradoxically, OCT4 mRNA can at least be derived into OCT4A, OCT4B and OCT4B1 transcript variants being upon mRNA alternative splicing. Of course, only OCT4A spliced mRNA is constituted of exon1. It is finally translated to produce OCT4A isoform in which the N-terminal has been derived from exon1. Despite the recent evidence, this translational product endows its pivotal role in actively maintaining the self-renewal. In the meantime, the molecular composites of the other OCT4 spliced variants do not contain exon1 as well as the N-termini of their translated isoforms; OCT4B and OCT4B1, have been decoded from exon2 of their corresponding OCT4 spliced mRNA. Since cancer stem cells could be considered as an aberrant caricature of embryonic stem cells; therefore, the manipulation of their self-renewal is more likely analogous to its counterpart, embryonic stem cells (ESC) and it would be achieved through the expression of OCT4A spliced mRNA. Therefore, its functional translated protein OCT4A is assumptively exert as the key component in the regulatory axis. Consequently, the detection of the expression of OCT4A spliced variant and OCT4A in any CSC ought to be the trusty convenient strategy for characterizing the putative cancer stem cells in all neoplastic diseases.

Pathophysiologically, individual viable cells including neoplastic cells can cope with various alterations of intrinsic and/or extrinsic microenvironments differently, by changing their biochemical functionality across the time. This biological issue results in the variances of cellular characteristics when measured at any timepoint, referred to cellular heterogeneity. This instability is influenced by genetic and epigenetic elements, microenvironments, cell-to-cell communications, and/or cell to-acellular component interactions in a variety of responsible pathways. Thence, cellular heterogeneity can confuse the real-time intricate biology of studied cells leading to unsustainable data interpretation. Particularly, when the conventional methods, such as immunohistochemistry (IHC), polymerase chain reaction (PCR), reverse transcription PCR (RT-PCR) and microscopic morphometry are used since each of them is grossly measuring an average biological signal from all mixed cell subpopulations. Indubitably, the strongest signal from the major subpopulation can frequently interfere with the weaker signals which are significantly generated by the etiologic cell groups, for example, cancer stem cells in many neoplastic subjects. Thus, the surrogate signal may not reflect the real biology of targeted cells unless no plasticity of causative cells.

This supplementary provides the preliminary result of OCT4A RT-PCR done with a batch of specific OCT4A. The sequences of the primers consist of the forward; 5′-CTT CCG ACT TGG CCT TCT-3′ and the reverse; 5′-AAG GAG AAG CTG GAG CAA-3′. This primers set was established by our laboratory to detect the exon 1 in OCT4A transcript variant. As predicted, 405 bp-amplicon of exon 1 was amplified by both conventional PCR and revere transcription PCR. Howsoever, only 1 out of 6 specimens exhibited the expression of OCT4A splice variant based on RT-PCR. Intuitively, all controls were negative, indicating there were no self-renewing cells actively functioning in those specimens. This has implied that not all canine cutaneous mast cell tumor (MCT) cells in those specimens had the same capacity to self-renew. Moreover, they might have the distinguished bioactivities over the time, especially OCT4A expression. Thence, if those cells were the same, they should exhibit the similar biology in all specimens based on the traditional cancer model. But, the preliminary result has suggested the cellular heterogeneity instead.

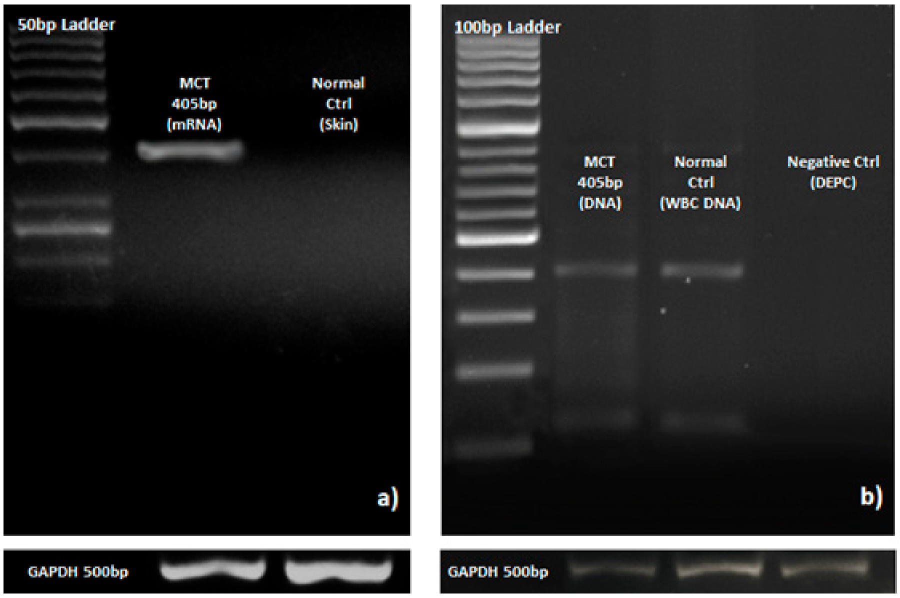


**Figure S1.** The 405 bp amplicons of OCT4A. (**a**) Two-step RT-PCR exhibits the expression of OCT4A in MCT specimens, when compared to normal dog skin. (**b**) Conventional PCR products of OCT4A amplified from the DNA templates of MCT cells and canine white blood cells (WBC), respectively. The result has been used to confirm the specificity of OCT4A primers. .
